# Supplementary material for: Reconciling Mining with the Conservation of Cave Biodiversity: A Quantitative Baseline to Help Establish Conservation Priorities
Source: PLoS One. 2016 Dec 20;11(12):e0168348. doi: 10.1371/journal.pone.0168348 (PMC5173368; doi:10.1371/journal.pone.0168348)
Supplement: S1 Dataset — (ZIP) [file pone.0168348.s002.zip › Taxa/Serra Norte/SN_2013/N4E/Lista N4E-06.pdf]

CAVIDADE N4E-0006

Classe	Ordem	Fam/Outros	Gên/Outros	Espécie	S-U	U-S	Zon.
Arachnida	Acari	(Oribatida)		sp.3		X	E
Arachnida	Acari	(Parasitengonina)		sp.12		X	P
Arachnida	Acari	Ixodidae	*Amblyomma*	*cajennense*	X		
Arachnida	Acari	Mesostigmata		sp.42		X	P
Arachnida	Acari	Neothyridae	*Diplothyrus*	jovem		X	P
Arachnida	Araneae	Corinnidae		jovem	1	1	P
Arachnida	Araneae	Ctenidae		jovem	1	1	P
Arachnida	Araneae	Mysmenidae		jovem		X	E/P
Arachnida	Araneae	Ochyroceratidae		sp.	X	X	P
Arachnida	Araneae	Oonopidae	aff. *Xyccarph*	sp.1	X		
Arachnida	Araneae	Pholcidae	*Leptopholcus*	sp.1		X	P
Arachnida	Araneae	Pholcidae	*Mesabolivar*	sp.1	X	X	P
Arachnida	Araneae	Pholcidae	*Metagonia*	sp.	X		
Arachnida	Araneae	Pholcidae	Ninetinae	sp.1		X	P
Arachnida	Araneae	Prodidomidae		jovem		X	E
Arachnida	Araneae	Salticidae		sp.	X		
Arachnida	Araneae	Segestriidae	*Ariadna*	sp.1		X	P
Arachnida	Araneae	Theridiosomatidae	*Ogulnius*	sp.1		X	E
Arachnida	Araneae	Uloboridae	*Uloborus*	sp.1		X	E
Arachnida	Opiliones	Cosmetidae		sp.	X		
Arachnida	Opiliones	Manaosbiidae	*Saramacia*	sp.1		1	P
Arachnida	Palpigradi	Eukoeneniidae	*Eukoenenia*	sp.1		X	P
Arachnida	Pseudoscorpiones	Chernetidae	*Spelaeochernes*	sp.1	X	X	E/P
Arachnida	Pseudoscorpiones	Chthoniidae	*Pseudochthonius*	sp.1		X	E/P
Chilopoda	Scolopendromorpha	Scolopocryptopidae	*Newportia*	sp.2		1	P
Chilopoda	Scolopendromorpha	Scolopocryptopidae	*Tidops*	jovem		1	E
Chilopoda	Scutigeromorpha	Pselliodidae	*Sphendononema*	*guildingii*	X		
Diplopoda	Polydesmida	Chelodesmidae		jovem		1	E/P
Diplopoda	Polydesmida	Fuhrmannodesmidae		jovem		X	P
Diplopoda	Polydesmida	Pyrgodesmidae		sp.1	X		
Entognatha	Collembola	Entomobryidae		sp.6		X	P
Entognatha	Collembola	Isotomidae		sp.1		X	P
Entognatha	Collembola	Paronellidae		sp.1		X	E/P
Entognatha	Collembola	Paronellidae		sp.4		X	P
Entognatha	Collembola	Poduromorpha		sp.1		X	E
Entognatha	Collembola			sp.	X		
Entognatha	Diplura	Campodeidae		sp.1		X	E/P
Entognatha	Diplura	Projapygidae		sp.1		X	E/P
Insecta	Blattodea			jovem	X	2	P
Insecta	Coleoptera	Elateridae		jovem	X		
Insecta	Coleoptera	Staphylinidae		sp.1		X	P
Insecta	Coleoptera	Staphylinidae		sp.58		X	E
Insecta	Diptera	Bombyliidae		sp.		X	E
Insecta	Diptera	Cecidomyiidae	Cecidomyiinae	sp.		X	P
Insecta	Diptera	Ceratopogonidae		sp.		X	P
Insecta	Diptera	Psychodidae	Phlebotominae	sp.		X	E/P
Insecta	Diptera	Tipulidae	Tipulinae	sp.		X	P
Insecta	Heteroptera	Nabidae	*Arachnocoris*	jovem		X	E
Insecta	Heteroptera	Reduviidae	Emesinae	sp.6		2	P
Insecta	Heteroptera	Reduviidae	*Zelurus*	sp.	X	2	P
Insecta	Homoptera	Cixiidae		jovem	X	X	P
Insecta	Homoptera	Cixiidae		sp.2		X	P
Insecta	Homoptera	Cixiidae		sp.3		X	P
Insecta	Homoptera	Cixiidae		sp.5		X	P
Insecta	Hymenoptera	Formicidae	*Camponotus*	*atriceps*	X		

Insecta	Hymenoptera	Formicidae	*Camponotus*	sp.1		X	E/P
Insecta	Hymenoptera	Formicidae	*Crematogaster*	sp.1		X	P
Insecta	Hymenoptera	Formicidae	*Crematogaster*	sp.2		X	P
Insecta	Hymenoptera	Formicidae	*Dolichoderus*	aff. *bispinosus*		X	E/P
Insecta	Hymenoptera	Formicidae	*Eurhopalothrix*	sp.1		X	E
Insecta	Hymenoptera	Formicidae	*Gnamptogenys*	sp.1		X	E
Insecta	Hymenoptera	Formicidae	*Hypoponera*	sp.1		X	P
Insecta	Hymenoptera	Formicidae	Myrmicinae	sp.1		X	P
Insecta	Hymenoptera	Formicidae	*Pachycondyla*	sp.2		X	P
Insecta	Hymenoptera	Formicidae	*Pheidole*	sp.1		X	E
Insecta	Hymenoptera	Formicidae	*Pheidole*	sp.2		X	E/P
Insecta	Hymenoptera	Formicidae	*Trachymyrmex*	sp.1		X	P
Insecta	Isoptera	Termitidae	*Embiratermes*	sp.		X	P
Insecta	Isoptera	Termitidae	*Nasutitermes*	sp.		X	P
Insecta	Isoptera	Termitidae		sp.	X		
Insecta	Lepidoptera	Noctuoidea		sp.7		1	P
Insecta	Lepidoptera	Tineoidea		jovem	X		
Insecta	Neuroptera	Myrmeleontidae		jovem		X	P
Insecta	Orthoptera	Gryllidae		jovem		1	P
Insecta	Orthoptera	Phalangopsidae	*Paraclodes*	sp.	X		
Insecta	Psocoptera			jovem	X	X	E/P
Insecta	Thysanura	Nicoletiidae		sp.1		X	E/P
Malacostraca	Isopoda	Armadillidae		sp.1		X	P
Pauropoda	Tetramerocerata			jovem		X	E
Symphyla		Scutigerellidae		sp.		X	E/P
Amphibia	Anura			sp.	1	2	E/P
Mammalia	Chiroptera	Emballonuridae	*Peropteryx*	sp.	4	1	P
Mammalia	Rodentia	Cricetidae	*Rhipidomys*	sp.		1	P
